# Integrated Prediction Framework for Clinical Scores of Cognitive Functions in ESRD Patients

**DOI:** 10.1155/2022/8124053

**Published:** 2022-08-09

**Authors:** Yutao Zhang, Quan Sheng, Xidong Fu, Haifeng Shi, Zhuqing Jiao

**Affiliations:** ^1^School of Microelectronics and Control Engineering, Changzhou University, Changzhou 213164, China; ^2^School of Computer Science and Artificial Intelligence, Changzhou University, Changzhou 213164, China; ^3^Department of Radiology, Changzhou Second People's Hospital Affiliated to Nanjing Medical University, Changzhou 213003, China

## Abstract

The clinical scores are applied to determine the stage of cognitive function in patients with end-stage renal disease (ESRD). However, accurate clinical scores are hard to come by. This paper proposed an integrated prediction framework with GPLWLSV to predict clinical scores of cognitive functions in ESRD patients. GPLWLSV incorporated three parts, graph theoretic algorithm (GTA) and principal component analysis (PCA), whale optimization algorithm with Levy flight (LWOA), and least squares support vector regression machine (LSSVRM). GTA was adopted to extract features from the brain functional networks in ESRD patients, while PCA was used to select features. LSSVRM was built to explore the relationship between the selected features and the clinical scores of ESRD patients. Whale optimization algorithm (WOA) was introduced to select better parameters of the kernel function in LSSVRM; it aims to improve the exploration competence of LSSVRM. Levy flight was used to optimize the ability to jump out of local optima in WOA and improve the convergence of coefficient vectors in WOA, which lead to an increase in the generalization ability and convergence speed of WOA. The results validated that the prediction accuracy of GPLWLSV was higher than that of several comparable frameworks, such as GPSV, GPLSV, and GPWLSV. In particular, the average of root mean square error (RMSE), mean absolute error (MAE), and mean absolute percentage error (MAPE) between the predicted scores and the actual scores of ESRD patients was 2.40, 2.06, and 9.83%, respectively. The proposed framework not only can predict the clinical scores more accurately but also can capture imaging markers associated with decline of cognitive function. It helps to understand the potential relationship between structural changes in the brain and cognitive function of ESRD patients.

## 1. Introduction

In recent years, the mortality rate of patients with end-stage renal disease (ESRD) has been increasing year by year. ESRD is the second highest increase of any disease, which is one of the top risks to human health. Although ESRD patients represent 0.1% of the global population, World Public Health Organization spends 2%-3% of its total expenditure on the treatment of ESRD. ESRD not only places a severe economic burden on society and families but also places a significant mental burden on patients [1]. Cognitive impairment is common in ESRD patients, especially in the aspects of orientation, attention, and executive ability [2]. Bugnicourt et al. [3] found that the incidence of cognitive impairment in patients undergoing hemodialysis was as high as 30% ∼ 60%. Cognitive impairment may affect late treatment of ESRD patients, such as dietary adjustment and medication compliance. Cognitive impairment in ESRD patients is often ignored in clinical practice, and its pathophysiological mechanism has not been fully elucidated [4]. In consequence, it is of great significance to study the cognitive impairment mode of patients with ESRD and clarify the exact pathophysiological mechanism.

As neuroimaging technology develops by leaps and bounds, the neuropathological mechanism of ESRD can be learned from the perspective of central nervous systems. For example, Liang [5] et al. observed the brain neural activity of ESRD patients with the help of resting-state fMRI technology. Compared with normal controls, the functional activity of ESRD patients significantly decreased in bilateral frontal parietal temporal lobe, suggesting that the abnormal connection of brain functional networks in ESRD patients could result in cognitive disorders. In virtue of Diffusion Tensor Imaging (DTI) technology, Chou et al. [6] pointed out that long-term hemodialysis would aggravate cerebral interstitial edema in ESRD patients and cause demyelination of pontine axons, indicating that hemodialysis may lead to extensive white matter damage in the brain. Chai et al. [7] found that the volume of gray matter in the left lobe and bilateral putamen of ESRD patients was significantly lower than that of healthy subjects through voxel-based morphometry. This suggests that changes in the volume of gray matter in the left lobe bring about cognitive impairment probably. These researches relying on neuroimaging technology have greatly deepened our understanding of the cognitive function of ESRD patients, but it is still impossible to determine the stage of cognitive function of ESRD patients.

Clinically, neurologists often judge the stage of cognitive function of ESRD patients according to the scores of the Montreal Cognitive Assessment Scale (MoCA), also known as clinical scores. Accurate prediction of clinical scores is beneficial to estimating the stage of cognitive functions of ESRD patients. Jiang et al. [8] calculated the correlation coefficient between topological attribute parameters of brain functional networks and clinical scores of cognitive functions in ESRD patients by Pearson correlation analysis. They mainly focused on the imaging markers affecting the cognitive function of ESRD patients and could not judge the current status of cognitive function well. Lu et al. [9] proposed a prediction method of clinical scores based on the brain functional networks. By virtue of simple definition and small computation, clustering coefficients in the brain functional networks were extracted as features to predict clinical scores, which were referred to judge the current stage of cognitive function. However, this method is subjective in the process of extracting features; at the same time, the influence of other features on the cognitive function of patients is ignored. Yang et al. [10] predicted clinical scores by support vector regression machine (SVRM). Regrettably, SVRM has high volatility and low prediction accuracy in the process of predicting clinical scores.

On the above considerations, we propose an integrated framework to predict clinical scores of ESRD patients. The main work is as follows: firstly, fMRI data were preprocessed to construct the brain functional networks, and graph theoretic algorithm (GTA) was adopted to extract features. Secondly, principal component analysis (PCA) was used to select features. Then, the least squares support vector regression machine (LSSVRM) was built to explore the relationship between the selected features and the clinical scores of ESRD patients. Meanwhile, the whale optimization algorithm (WOA) was introduced to select better parameters of kernel function in LSSVRM so as to enhance the exploration competence of LSSVRM. Finally, Levy flight replaced the traditional selection of WOA and in the meantime optimized the ability to jump out of local optimum in WOA. The framework called GPLWLSV was constructed to predict the clinical scores of ESRD patients and then determine their current stage of cognitive function.

## 2. Data and Methods

### 2.1. Research Framework

**Figure 1:**
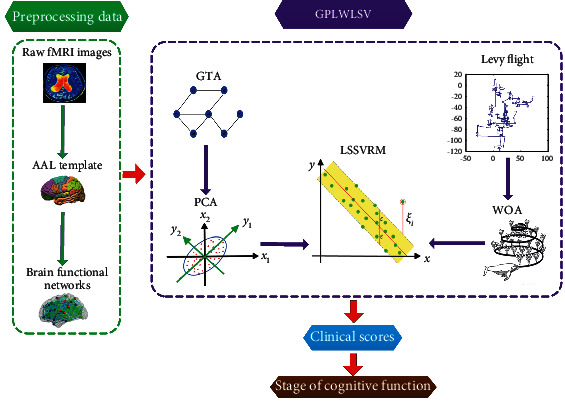
A flowchart of prediction framework.

Figure 1 shows our prediction framework, which mainly includes the following steps: (1) The original resting-state fMRI images were preprocessed by Data Processing Assistant for Resting-State fMRI (DPARSF). (2) Time series from preprocessed images were abstracted to construct the brain functional networks. (3) The area under the curve (AUC) of topological attribute parameters in the brain functional networks was extracted as features through GTA. (4) PCA was used to filter redundant features and to retain important features. (5) LSSVRM was built to retain features. (6) WOA was improved by Levy flight to optimize the selection strategy of kernel function parameters in LSSVRM. (7) GPLWLSV was constructed to predict clinical scores of cognitive functions in ESRD patients.

### 2.2. Experimental Data and Pretreatment

A total of 50 patients with ESRD were admitted to Changzhou Second People's Hospital Affiliated to Nanjing Medical University from May 2021 to March 2022, including 27 male and 23 female individuals aged 49.12 ± 8.23 years. Synchronously, a total of 40 normal controls were also admitted to the same hospital, including 22 male and 18 female individuals aged 47.26 ± 7.01 years. There were no significant differences (*P* > 0.05) in the gender ratio, age, and education level between them. One hour before fMRI examination, the stage of cognitive function of all subjects was assessed by trained neurologists who did not know the data of subjects via clinical scores of cognitive functions.  Table 1 gives the demographic information of these two groups of subjects.

All subjects were scanned with GE Discovery MR 750W 3.0T superconducting MR scanner with 32-channel head and neck joint coil. The head of subject was fixed with a cushion to reduce the artifacts produced by head movement. Gradient echo plane echo imaging (GRE-EPI) sequence was used to collect fMRI images. During the collection process, subjects were required to keep quiet and awake and try not to think. Machine scanning parameters are as follows: repetition time (TR) = 2000 ms, echo time (TE) = 40 ms, field of view (FOV) = 24 cm, flip angle (FA) = 90°, matrix size = 64 × 64, and layer thickness = 6 mm.

After achieving the fMRI images of all subjects, DPARSF (available at http://rfmri.org/dpabi) participated in preprocessing. The specific steps are as follows: (a) Image format was transformed: the correct format can only be opened by DPARSF. (b) The first 10 time points were deleted: errors may occur when the instrument and subjects were not in a stable state. (c) Time points were corrected: data of time points at different levels were corrected to the same time point to maintain consistency. (d) Brain positions were corrected: the brain must remain in the same position throughout the examination. (e) Space was normalized: put the brain of subject into a standard space for the convenience of follow-up statistics and reports. (f) Spatial structure differences were reduced through smooth: better image results were beneficial to the validity of statistical tests. (g) Linear drift was removed: changes in time and temperature caused errors in the inspection instrument. (h) Filtering was performed: the frequency range is 0.01∼0.08 Hz. The resting fMRI signal after low-frequency filtering has important physiological significance and may reflect spontaneous neural activity. (i) Final time series was obtained: the mean BOLD time series of head motion parameters, white matter, and cerebrospinal fluid were regression and removed.

### 2.3. Principle of GP

#### 2.3.1. Features Were Extracted by GTA

The construction of brain functional networks is as follows. Firstly, the brain of each subject was divided into 90 brain regions on the basis of Automated Anatomical Labeling (AAL). Secondly, the Pearson correlation coefficient was calculated between the time series of two brain regions; **K**-matrix was born, which is a 90 × 90 symmetric matrix with all 1 s on the diagonal. Finally, the Fisher-Z transformation is performed on the elements in the **K**-matrix divided by the diagonal, which is converted to the Z-value close to the normal distribution to generate the **Z**-matrix:

(1) $$\begin{array}{c} \text{K}=\left(\begin{array}{cccc} 1 & p_{\left(1,2\right)} & \cdots & p_{\left(1,90\right)}\\ p_{\left(2,1\right)} & \ddots & \; & \vdots \\ \vdots & \; & \ddots & \vdots \\ p_{\left(90,1\right)} & \cdots & \cdots & 1 \end{array}\right), \end{array}$$

(2) $$\begin{array}{c} \text{Z}=\left(\begin{array}{cccc} 1 & z_{\left(1,2\right)} & \cdots & z_{\left(1,90\right)}\\ z_{\left(2,1\right)} & \ddots & \; & \vdots \\ \vdots & \; & \ddots & \vdots \\ z_{\left(90,1\right)} & \cdots & \cdots & 1 \end{array}\right), \end{array}$$

(3) $$\begin{array}{c} z_{\mathrm{ij}}=\frac{1}{2}\left[\ln\left(1+p_{\mathrm{ij}}\right)-\ln\left(1-p_{\mathrm{ij}}\right)\right]\left(i\neq j\right), \end{array}$$

where *p*_*ij*_ represents the Pearson correlation coefficient between the time series of the *i*-th brain region and the *j*-th brain region.

Taking matrix sparsity as the threshold, the preprocessing for **Z**-matrix was binary. The matrix sparsity was set to 0.1–0.4 with a span of 0.01. Within the threshold range of the matrix sparsity, the AUC of topological attribute parameters was calculated by GTA as features, which reflected the overall change of brain functional networks and reduced the influence of individual differences. These features include the AUC of global efficiency (Eglobal), local efficiency (Elocal), clustering coefficient (Cp), characteristic path length (Lp), standardized clustering coefficient (*γ*), standardized characteristic path length (*λ*), and small-world properties (*σ*).

#### 2.3.2. Features Were Selected by PCA

In the process of machine learning, a large number of features need to be extracted and converted into various data that can be processed by computers [11]. Increasing the number of features can improve the effect of machine learning. The trouble is that as the number of features increases, the dimension of feature vectors will also add. This not only makes machine learning more difficult but can also lead to overfitting, affecting the final accuracy. In view of this situation, it is necessary to filter out some unimportant features or combine some related features [12]. Features were selected by PCA that keep the information contained in the original data as much as possible while reducing the dimension. The specific steps are as follows.

The first step is to standardize the matrix. The AUC of Eglobal, Elocal, Cp, Lp, *γ*, *λ*, and *σ* of ESRD patients was marked as *α*1∼*α*7. Constructing a matrix called *A*, *A* = [*α*1, *α*2, *α*3, *α*4, *α*5, *α*6, *α*7], and *α*_*j*_ = [*x*_1*j*_, *x*_2*j*_, *x*_3*j*_…… *x*_*nj*_]*T*.

B-matrix is the normalization of A-matrix, *B* = [*β*1, *β*2, *β*3, *β*4, *β*5, *β*6, *β*7], and *β*_*j*_ = [ *y*_1*j*_, *y*_2*j*_, *y*_3*j*_…… *y*_*nj*_] *T* [13]:

(4) $$\begin{array}{c} y_{\mathrm{ij}}=\frac{x_{\mathrm{ij}}-\bar{x}}{\sqrt{\left({\left(x_{\mathrm{ij}}-\bar{x}\right)}^{2}/\left(n-1\right)\right)}}, \end{array}$$

where $\bar{x}$ is the mean value of samples in *α*_*j*_ and *n* is the number of samples.

The second step is to construct the covariance matrix of **B**-matrix. Computing the covariance between *β*_*i*_ and *β*_*j*_ in *β*_1_ ∼ *β*_7_,

(5) $$\begin{array}{c} r_{\mathrm{ij}}=\mathrm{cov}\left(X,Y\right)\\ =\frac{\sum_{i=1}^{n}\left(X_{i}-\bar{X}\right)\left(Y_{i}-\bar{Y}\right)}{n-1}, \end{array}$$

where *X*_*i*_ is the value of the *i*-th sample; $\bar{X}$ represents the mean value of samples; *n* is the number of samples.

Constructing the covariance matrix called **R**,

(6) $$\begin{array}{c} \text{R}=\left(\begin{array}{cccc} r_{11} & r_{12} & \cdots & r_{1m}\\ r_{21} & \ddots & \; & r_{2m}\\ \vdots & \; & \ddots & \vdots \\ r_{m1} & r_{m2} & \cdots & r_{mm} \end{array}\right), \end{array}$$

where *m* represents the number of features and its value is 7.

The third step is to extract the original features. Computing the eigenvalues and eigenvectors of the **R**-matrix, the principal component is the eigenvector of the **R**-matrix, and the magnitude of the corresponding eigenvalue indicates the importance of the eigenvector. Calculating the weight of the features, *λ*_1_∼*λ*_7_ are the eigenvalues of the **R**-matrix, *λ*_*j*_ represents the dominance of ***α***_j_ in the **A**-matrix, and the weight of ***α***_j_ in the whole feature set called *α*_1_∼*α*_7_ can be expressed as [14]

(7) $$\begin{array}{c} w_{i}=\frac{\lambda_{i}}{\sum_{j=1}^{7}\lambda_{j}}. \end{array}$$

The weight of extracted features is higher than 0.6 set in this paper.

Finally, the extracted features are transformed to new features. Finding the eigenvector *v*_*j*_ in the **R**-matrix corresponding to *α*_*j*_, the new feature *c*_*j*_ is transformed from *v*_*j*_ [15]:

(8) $$\begin{array}{c} {\text{c}}_{j}=\text{B}\cdot {\text{v}}_{j}. \end{array}$$

### 2.4. Principle of LSSVRM

The principle of SVRM is to map data vectors from low-dimensional space to high-dimensional space. SVRM constructs decision functions with the aid of the principle of risk minimization. SVRM is suited to solve nonlinear problems of small samples and high dimension of feature vectors. LSSVRM is simplified on the basis of SVRM. There are two main points: (1) changing constraints in SVRM to improve computing efficiency; (2) selecting different decision functions to reduce the operation time. LSSVRM maps nonlinear vector Φ(*x*) to high-dimensional space and transforms it into a linear regression problem, as shown in formula (9) [16]:

(9) $$\begin{array}{c} y=\omega^{T}\Phi \left(x\right)+b, \end{array}$$

where *ω* is the n-dimensional weight vector; *b* is the deviation.

Following the principle of risk minimization, linear regression can be transformed into an optimal problem [17]:

(10) $$\begin{array}{c} \min J\left(\omega ,\xi \right)=\frac{1}{2}\omega^{T}\omega +\frac{1}{2}J\sum_{i=1}^{m}\xi_{i}^{2}, \end{array}$$

(11) $$\begin{array}{c} y_{i}=\omega^{T}\Phi \left(x_{i}\right)+b+\xi_{i}, \end{array}$$

where *J* represents the normalization function; *ξ*_*i*_ represents the error; *i* represents the *i*-the dimension of the space vector; *ω* represents the weight vector. Since *ω* belongs to the high-dimensional space, it cannot be solved directly, so the kernel function is introduced [18]:

(12) $$\begin{array}{c} y=\sum_{i=1}^{n}a_{i}K\left(x,x_{i}\right)+b. \end{array}$$

In order to ensure the efficiency of operation, RBF is chosen as the kernel function, and its function is expressed as follows [19]:

(13) $$\begin{array}{c} K\left(x_{i},x_{j}\right)=\exp\left(-\frac{\left||x_{i}-x_{j}|\right|}{2\sigma^{2}}\right). \end{array}$$

It can be seen from the above process that *J* and *σ*^2^ have the greatest influence on the LSSVRM. In searching for optimal *J* and *σ*^2^, WOA is replaced by LWOA to seek the optimal solution.

### 2.5. Principle of LWOA

WOA is a new optimization algorithm that simulates the predatory behavior of whales. Whales locate and surround prey through their scent. We can define a certain number of virtual whales as search agents, assuming that the location of the scent of prey is at or near the current optimal location. We look for the optimal solution as the next location of the whale by comparing the feasible solutions of various search agents. Other search agents update their location to complete the strategy of finding the optimal solution.

Whales constantly adjust their position according to the position of prey during hunting. To describe this strategy of hunting, the following mathematical model is presented [20]:

(14) $$\begin{array}{c} M_{1}=\left|CX^{*}\left(t\right)-X\left(t\right)\right|,\\ X\left(t+1\right)=X^{*}\left(t\right)-HM_{1}, \end{array}$$

where *t* is the number of current iterations; **X**(*t*) is the coordinate vector of the current whale; **X**(*t*+1) is the target coordinate vector after the next iteration; **X**^*∗*^(*t*) is the coordinate vector of the best solution at present. If there is a better feasible solution, **X**^*∗*^(*t*) should be updated immediately. C and H are coefficients, which are acquired by formulas (22) and (23) [21]:

(15) $$\begin{array}{c} H=2hr_{1}-h,\\ C=2r_{2}, \end{array}$$

where *r*_1_and *r*_2_ are random numbers between 0 and 1; *h* is computed from formula (24) [21]:

(16) $$\begin{array}{c} h=2-\frac{2t}{T_{\max}}. \end{array}$$

For purpose of finding a better target position to approach the prey, the whale will randomly use any whale coordinate vector to replace the whale coordinate vector of the next iteration so as to achieve the purpose of deviating from the prey. This avoids falling into local optimality. The following mathematical model is shown [22]:

(17) $$\begin{array}{c} X\left(t+1\right)=\left\{\begin{array}{c} X^{*}\left(t\right)-HM_{1},\;\left|H\right|<1,\;p<0.5,\\ X_{\mathrm{rand}}\left(t\right)-HM_{2},\;\left|H\right|⩾1,\;p<0.5,\\ X^{*}\left(t\right)+M_{3}e^{bl}\cos\left(2\pi l\right),\;p⩾0.5, \end{array}\right.\\ M_{1}=\left|CX^{*}\left(t\right)-X\left(t\right)\right|,\\ M_{2}=\left|CX_{\mathrm{rand}}-X\left(t\right)\right|,\\ M_{3}=\left|X^{*}\left(t\right)-X\left(t\right)\right|, \end{array}$$

where *t* is the current iteration times; **X**^*∗*^(*t*) is the optimal position vector so far; **X**_rand_ is the random position vector of whale; **X**(*t*) is the current position vector of whale; *b* is the constant, and default is 1; the role of *b* is to control the hunting path shape; *l* is acquired by the following formulas:

(18) $$\begin{array}{c} l=\left(h_{2}-1\right)r_{3}+1,\\ h_{2}=-1-\frac{t}{T_{\max}}, \end{array}$$

where *r*_3_ is a random number between 0 and 1; *t* represents the current iteration number; *T*_max_ represents the maximum iteration number. When *t* ≥ 0.5*T*_max_, H is always less than 1. At this point, the whale enters the attack mode and no longer deviates from the prey through search agents.

Due to the few adjustment parameters of WOA, it has a fast rate of convergence and a certain ability to jump out of the local optimum. It is worth noting that WOA can be optimized further. The reasons are as follows: Firstly, WOA searches by means of random system. Excessive reliance on random system limits the convergence speed of WOA. Secondly, WOA is subject to coefficient vector. WOA will lose the ability to jump out of the local optimum when the number of iterations reaches half of the maximum number of iterations set earlier. Consequently, WOA is accompanied by a certain risk of falling into the local optimum, leading to inaccurate results of prediction [23].

The defects of WOA can be solved by an improved WOA with Levy flight named LWOA. Levy flight is a kind of random search that relies on Levy distribution, which has been applied many times in the optimization field in recent years. Levy flight is able to improve cuckoo and particle swarm optimization algorithms [23–25] and so on. LWOA owns a faster convergence speed and higher convergence accuracy; LWOA has a better ability to jump out of the local optimum. The specific steps of improvement are as follows.

WOA is improved by Levy flight, and formula (22) is replaced by the following formula [16, 26]:

(19) $$\begin{array}{c} H=2h\text{Levy}\left(\lambda \right)-h, \end{array}$$

where Levy(*λ*) means that it obeys the Levy distribution with parameter *λ* [16, 27]:

(20) $$\begin{array}{c} \text{Levy}∼\mu =t^{-\lambda }. \end{array}$$

Due to the complexity of Levy flight, the Mantegna algorithm is adopted to simulate it, and its mathematical expression is as follows [16, 28]:

(21) $$\begin{array}{c} s=\frac{\mu }{{\left|\nu \right|}^{\left(1/\beta \right)}}, \end{array}$$

where *μ* and *v* obey the normal distribution with parameters *σ*_*μ*_ and *σ*_*v*_ [16, 29]:

(22) $$\begin{array}{c} \mu ∼N\left(0,\sigma_{\mu }^{2}\right), \end{array}$$

(23) $$\begin{array}{c} \sigma_{\mu }=\left\{\frac{\Gamma \left(1+\beta \right)\sin\left(\pi \beta /2\right)}{\Gamma \left(1+\beta /2\right)\beta 2^{\left(\beta -1/2\right)}}\right., \end{array}$$

(24) $$\begin{array}{c} \sigma_{\nu }=1. \end{array}$$

For higher operation efficiency of the algorithm, *β* is a constant 1.5, and *σ*_*μ*_ is a constant 0.7.

The coefficient vector **H** in WOA converges linearly with certain limitations. WOA should be promoted to jump out of local optimum; formula (24) changed by the following formula [16]:

(25) $$\begin{array}{c} h=2e^{0.15\left(-\log\;{\left(10t/T_{\max}\right)}^{4}\right)}, \end{array}$$

where *t* represents the current iteration number and *T*_max_ represents the maximum iteration number.

In the early stage of iteration, the value of *h* will decrease slowly as the increase of iteration times, which is conducive to the global search out of local optimum. At the end of iteration, the value of *h* will decrease exponentially to improve the ability of rapid local search.

## 3. Results

### 3.1. Experimental Settings

Table 2 shows the AUC of topology attribute parameters of the brain functional networks of ESRD patients and normal controls calculated by GTA. Within the whole matrix sparsity threshold range, the AUC of *γ*, *σ*, and Elocal in ESRD patients was significantly lower than that in normal controls, with statistical significance (*P* < 0.05). Nevertheless, there were no significant differences (*P* > 0.05) in the AUC of *λ*, Cp, Lp, and Eglobal.

Table 3 shows the corresponding weight of each feature in the feature set. The proportion of AUC of Elocal was the highest, up to 65.31%. The proportion of AUC of Eglobal was next to Elocal, accounting for 30.89%. The proportion of AUC of *γ*, *λ*, *σ*, Cp, and Lp was less than 5%. The proportion of *γ* was the lowest, only 0.03%.

The experimental results can be seen from Tables 2 and 3. The AUC of Elocal in ESRD patients was significantly lower than that in the normal controls, with statistically significant differences (*P* < 0.05). Meanwhile, the AUC of Elocal accounted for the highest proportion in the feature set. Obviously, the AUC of Elocal was selected as the feature to construct GPSV [30], GPLSV [31], GPWLSV [32], and GPLWLSV for predicting the clinical scores of cognitive functions in ESRD patients [33].

The prediction accuracy of various frameworks was evaluated by a tenfold cross-validation method. Firstly, the AUC of Elocal of 50 ESRD patients and the corresponding clinical scores were collected as data set called *D*. Secondly, *D* was divided into 10 mutually exclusive subsets of the same size, *D* = *D*_1_∪*D*_2_∪ *D*_3_∪*D*_4_∪*D*_5_∪*D*_6_∪*D*_7_∪*D*_8_∪*D*_9_∪*D*_10_, and *D*_i_∪*D*_j_ = ∅. Each subset *D*_i_ was separated from *D* through stratified sampling, which ensures consistency of data distribution. Each subset *D*_i_ contains five samples. Taking the union of 9 subsets as the training set and the remaining subset as the test set, 10 groups of training sets and test sets are formed. Different frameworks were trained and tested for 10 times, and the average of 10 groups of test results was calculated.

### 3.2. Experimental Results

**Figure 2:**
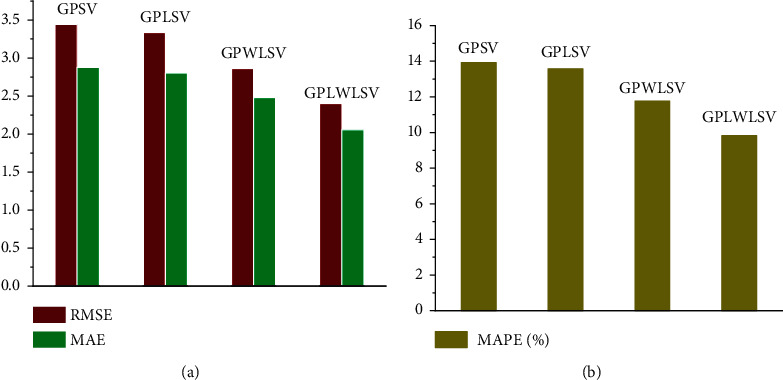
Average of prediction accuracies of various frameworks. (a) The RMSE and MAE of various frameworks. (b) The MAPE of various frameworks.

The root mean square error (RMSE), mean absolute error (MAE), and mean absolute percentage error (MAPE) were selected as the testing standards of prediction accuracies.  Table 4 shows the prediction accuracies of some representative frameworks. All the comparable predictive frameworks were evaluated by tenfold cross-validation method. The test results show that the prediction accuracy of GPLWLSV is higher than that of GPSV, GPLSV, and GPWLSV. The average of RMSE of GPLWLSV was 2.40, 1.04, 0.93, and 0.46 points lower than that of GPSV, GPLSV, and GPWLSV, respectively. The average of MAE of GPLWLSV was 2.06, which was 0.82, 0.74, and 0.42 points lower than that of GPSV, GPLSV, and GPWLSV, respectively. MAPE can reflect the relative errors of frameworks better than MAE. The average of MAPE of GPLWLSV was 9.83%, lower than 10%, which was 4.10%, 3.75%, and 1.93% lower than that of GPSV, GPLSV, and GPWLSV, respectively. The bar chart in Figure 2 shows that the prediction accuracy of GPLWLSV is better than that of GPSV, GPLSV, and GPWLSV intuitively.

**Figure 3:**
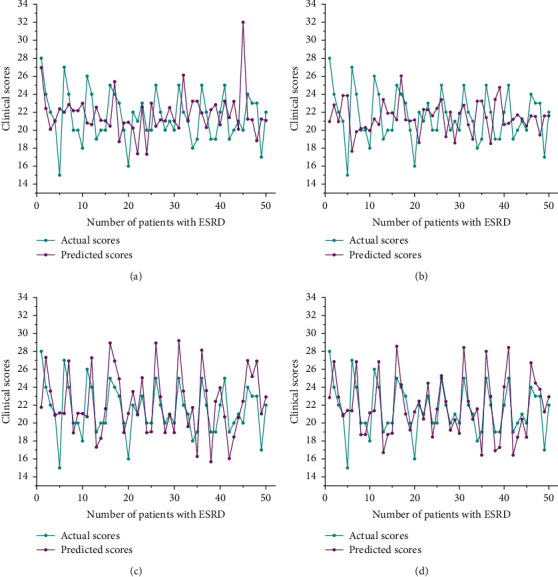
Actual scores and predicted scores of various frameworks. (a) GPSV, (b) GPLSV, (c) GPWLSV, and (d) GPLWLSV.

Figure 3 shows the comparison between the predicted scores of various frameworks and the actual scores. The solid blue line represents the actual scores, and the solid purple line represents the predicted scores. As can be seen from the figure, different from GPSV and GPLSV, GPWLSV and GPLWLSV can fit most of the scores of tests set well, and the predicted scores are closer to the actual scores in the case of large fluctuation. The strong fluctuation of actual scores will lead to a large error between the actual scores and the predicted scores of GPSV and GPLSV, while the predicted scores of GPWLSV and GPLWLSV are stable relatively. This is due to the powerful optimization ability of WOA.

Specifically, WOA optimizes the penalty factor and kernel parameters of LSSVRM, and we only need to adjust two parameters to improve the generalization ability and prediction accuracy of LSSVRM. The prediction accuracy of GPLWLSV is higher than that of GPWLSV for two reasons. Firstly, WOA adjusts parameters through a random system, and excessive reliance on a random system limits GPWLSV. Secondly, WOA is subject to coefficient vector. When the number of iterations reaches half of the maximum set earlier, WOA will lose its ability to jump out of the local optimum. At this time, WOA may fall into local optimum to some extent, which will lead to inaccurate prediction results. LWOA replaces the random adjustment of WOA with Levy flight; LWOA improves the convergence mode of coefficient vector of WOA; LWOA optimizes the ability of WOA to jump out of local optimum and thus improves the generalization ability and convergence speed of WOA. As a result, the prediction accuracy of GPLWLSV is higher than GPWLSV.

### 3.3. Discriminative Brain Regions

**Figure 4:**
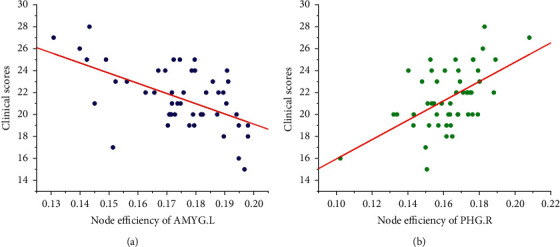
Relationship between node efficiency and clinical scores.

Node efficiency is one of the measures of node centrality. The higher the node efficiency is, the stronger the capacity of information transmission of the node is and the more important its position in the network is. The higher the efficiency of a node is, the stronger the information transmission capacity of the node is and the more important the node is in the network [34]. For the sake of finding out the key brain regions which affect the cognitive function of patients with ESRD [35], the multiple regression method was taken to analyze the relationship between the node efficiency and clinical scores of ESRD patients [36]. The results showed that the node efficiency of the left amygdala (AMYG.L) was negatively correlated with the clinical scores (*r* = −0.562, *P*=0.014) significantly, as shown in Figure 4(a), while the node efficiency of the right parahippocampal gyrus (PHG.R) was positively correlated with the clinical scores (*r* = 0.551, *P*=0.035) significantly, as shown in Figure 4(b). In addition, no correlation was found between node efficiency and clinical scores in other brain regions (*P* > 0.05). The amygdala is mainly involved in emotional processing [37], and previous studies have also shown that depression of varying degrees is common in patients with ESRD [38].

Given the above, it can be speculated that the amygdala node efficiency may be related to the depressed mood of ESRD patients, and the emotional instability and intense reaction of patients may lead to the increase of amygdala node efficiency. Qin et al. [39] found that patients with type 2 diabetes had multiple brain regions with increased node efficiency. This phenomenon is explained as the compensatory mechanism of the network. The right parahippocampal gyrus is associated with learning and memory functions [40], and its reduction of node efficiency may be connected with cognitive impairment in ESRD patients.

**Figure 5:**
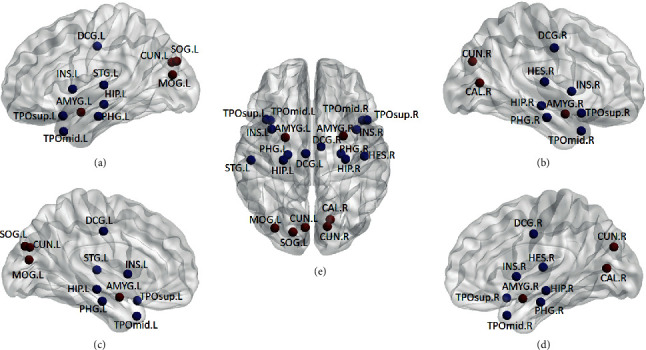
Brain regions with statistically significant differences in node efficiency between the two groups. (a) Left lateral view, (b) right lateral view, (c) left lateral view, (d) right lateral view, and (e) dorsal view of the whole brain.

To find out the relationship between each brain region and the cognitive function of ESRD patients, the node efficiency of 90 brain regions was calculated and contrasted *d* between the patients and the normal controls. The brain regions with significant differences were found; the result is shown in Figure 5. Blue nodes represent the brain regions with reduced node efficiency in ESRD patients compared to the normal controls. These included left insula (INS.L), right insula (INS.R), left median cingulate and paracingulate gyrus (DCG.L), right median and paracingulate gyrus (DCG.R), left hippocampus (HIP.L), right hippocampus (HIP.R), left parahippocampal gyrus (PHG.L), right parahippocampal gyrus (PHG.R), right transverse temporal gyrus (HES.R), left superior temporal gyrus (STG.L), left temporal pole: superior temporal gyrus (TPOsup.L), right temporal pole: superior temporal gyrus (TPOsup.R), left temporal pole: middle temporal gyrus (TPOmid.L), and right temporal pole: middle temporal gyrus (TPOmid.R). The red nodes represent the brain regions with increased node efficiency in ESRD patients compared to the normal controls. These include left amygdala (AMYG.L), right amygdala (AMYR.R), right calcarine fissure and surrounding cortex (CAL.R), left cuneus (CUN.L), right cuneus (CUN.R), left superior occipital gyrus (SOG.L), and left middle occipital gyrus (MOG.L).

The brain is a highly optimized complex system capable of integrating information to deal with changes in cognitive needs. According to research findings, the brain regions with reduced node efficiency in ESRD patients are mainly located in the paralimbic network. The hippocampus (HIP) and parahippocampal gyrus (PHG) are related to learning and memory functions [40]; meanwhile, the median and paracingulate gyrus (DCG) are involved in cognitive control function [41]. Chou et al. [42] applied DTI technology to view the changes in brain functional networks in ESRD patients, and the results also found that the node efficiency in the paralimbic network was significantly decreased compared with the normal controls. Notably, ESRD patients had higher node efficiency in brain regions associated with visual networks, which are responsible for processing visual information. These regions are about attention, visual memory, and other neurocognitive functions [43]. Accordingly, it can be speculated that the increased node efficiency in these brain regions may also be a compensatory mechanism [39]. In summary, node efficiency can effectively distinguish ESRD patients from the normal controls, thus achieving accurate classification.

## 4. Discussion

The purpose of this study was to look for imaging markers related to the decline of cognitive function in ESRD patients, which could be modeled to predict the clinical scores of the patients, and the clinical scores could determine the current stage of cognitive function. As a consequence, the GPLWLSV is constructed. The experimental results show that the GPLWLSV achieves the optimal effect of prediction than comparable frameworks, indicating its effectiveness. The GPLWLSV has three main advantages.

First of all, the imaging markers are accurate. By right of the principle of GP, GPLWLSV found the imaging markers related to the decline of cognitive function in ESRD patients. Compared with the normal controls, ESRD patients showed a significant decrease in the AUC of *γ*, *σ*, and Elocal at multiple sparsity. This result is consistent with a previous study based on resting-state fMRI networks [44]. *γ* and Elocal mainly affect specific information processing or fault tolerance rate of the network [45]. The decrease of *γ* and Elocal indicates that information transmission efficiency of different brain regions is reduced in ESRD patients, which impairs the ability to manage the brain potentially [46]. This provides a new perspective and potential imaging markers for understanding the underlying pathophysiological mechanisms of cognitive impairment in ESRD patients.

Secondly, the operation speed is faster. GPLSV has a shorter operation time than GPSV, although the prediction accuracy is similar. This is because LSSVRM is an improvement on SVRM. It changes inequality constraints into equality constraints in SVRM and transforms solving quadratic programming problems into solving linear equations, speeding up the operation speed greatly. In a word, the LSSVRM enables GPLWLSV to run more efficiently.

Finally, the prediction accuracy is higher. The prediction accuracy of GPWLSV and GPLWLSV is higher than that of GPSV and GPLSV significantly. This is because WOA possesses powerful optimization ability; it optimized the strategy of selecting two parameters in the kernel function of SVRM and LSSVRM, thus improving the prediction accuracy of GPWLSV and GPLWLSV. However, WOA still has deficiencies. Firstly, WOA adopts a random system to adjust parameters and relies on random system excessively. Secondly, WOA is limited by the coefficient vector. When the number of iterations reaches half of the maximum iterations set earlier, WOA will lose its ability to jump out of local optimum. In brief, WOA may fall into local optimum to some extent, leading to low prediction accuracy. GPLWLSV chose Levy flight instead of WOA to adjust parameters. LWOA improved the convergence mode of coefficient vector of WOA and hoisted the capacity of WOA to jump out of local optimum, and the convergence speed of WOA was improved. To sum up, the prediction accuracy of GPLWLSV was higher than comparable frameworks.

The prediction framework still has room for further improvement. The main reason is that the size of the data set in this paper is small, which affects the training effectiveness of GPLWLSV to a certain extent. Researches show that DTI, DWI, DKI, and other biomarkers of different modes are complementary to each other to some extent. Compared with single-mode analysis, multimode analysis can often achieve better results of prediction by integrating complementary information of different modes [47]. In future studies, more clinical data of different modes will be collected, leading to a more in-depth and comprehensive study on a larger data set.

## 5. Conclusion

In this paper, an integrated prediction framework with GPLWLSV was constructed to predict the clinical scores of cognitive functions in ESRD patients. According to the principle of GP, the framework found the imaging markers related to the decline of cognitive function in ESRD patients; meanwhile, GP determined that the AUC of Elocal accounted for the highest proportion of all the features. Elocal had a great influence on the cognitive function of ESRD patients. The inequality constraints in SVRM are simplified, and the operation speed is improved. LWOA was introduced to optimize the parameter selection strategy in LSSVRM, and the prediction accuracy was improved. In clinical diagnosis, it is often necessary to analyze the clinical scores of a large number of patients with ESRD in order to judge the stage of cognitive function. In general, our framework can obtain imaging markers related to the decline of cognitive function accurately and give consideration to work efficiency and accuracy simultaneously.

## Figures and Tables

**Table 1:** Demographic information of subjects.

	Gender (male/female)	Age (years, $\bar{x}\pm s$)	Education years (years, $\bar{x}\pm s$)	Clinical scores (points, $\bar{x}\pm s$)
ESRD patients (*n* = 50)	27/23	49.12 ± 8.23	11.13 ± 2.05	21.47 ± 2.75
Normal controls (*n* = 40)	22/18	47.26 ± 7.01	11.24 ± 2.13	27.38 ± 1.35
*t*/*χ*2	0.009	1.133	0.387	−13.412
*P*	>0.05	0.260	0.778	0.000

**Table 2:** Comparison of AUC of global topological parameters between two groups (mean ± SD).

Parameter	ESRD patients (*n* = 50)	Normal controls (*n* = 40)	*t*	*P*
*γ*	0.646 ± 0.071	0.669 ± 0.056	−1.714	0.004
*λ*	0.326 ± 0.123	0.329 ± 0.024	0.710	0.480
*σ*	0.589 ± 0.065	0.607 ± 0.065	−1.384	0.008
Cp	0.175 ± 0.014	0.176 ± 0.016	−0.296	0.768
Lp	0.543 ± 0.027	0.553 ± 0.069	−0.966	0.337
Eglobal	0.171 ± 0.006	0.170 ± 0.011	−0.477	0.635
Elocal	0.230 ± 0.007	0.241 ± 0.006	−0.253	0.001

**Table 3:** Weight of AUC of global topological parameters of ESRD patients in feature set.

Parameter	*γ* (%)	*λ* (%)	*σ* (%)	Cp (%)	Lp (%)	Eglobal (%)	Elocal (%)
*w*_*i*_ × 100%	0.03	0.07	0.17	0.28	3.25	30.89	65.31

**Table 4:** Prediction accuracies of comparable frameworks.

Prediction framework	The test group	RMSE	MAE	MAPE
GPSV	1	3.5064	2.3835	0.1364
GPSV	2	3.482	3.0951	0.1454
GPSV	3	3.2648	2.8499	0.1267
GPSV	4	3.6154	3.1792	0.1542
GPSV	5	2.5508	2.2965	0.1109
GPSV	6	2.2724	1.7919	0.079
GPSV	7	4.115	3.6825	0.1788
GPSV	8	2.8204	2.659	0.1277
GPSV	9	5.7247	4.0523	0.1999
GPSV	10	3.0707	2.7852	0.1334
Average		3.4423	2.8775	0.1392
GPLSV	1	5.2634	4.197	0.2149
GPLSV	2	4.6628	3.1899	0.1305
GPLSV	3	3.4819	2.8121	0.1491
GPLSV	4	3.1611	2.7837	0.1385
GPLSV	5	2.1046	1.8884	0.0898
GPLSV	6	2.162	2.1246	0.0994
GPLSV	7	3.3545	3.0199	0.1524
GPLSV	8	3.9891	3.725	0.18
GPLSV	9	2.2805	1.7733	0.0812
GPLSV	10	2.8879	2.4838	0.1214
Average		3.3348	2.7998	0.1357
GPWLSV	1	4.2408	3.4782	0.1695
GPWLSV	2	3.3293	2.8178	0.124
GPWLSV	3	3.0657	2.4178	0.1188
GPWLSV	4	3.3027	2.9839	0.1466
GPWLSV	5	1.3125	1.1388	0.0529
GPWLSV	6	1.9272	1.4114	0.0618
GPWLSV	7	2.9412	2.7196	0.1311
GPWLSV	8	2.7899	2.6865	0.1284
GPWLSV	9	2.6758	2.3282	0.1092
GPWLSV	10	3.0313	2.8014	0.1335
Average		2.8616	2.4784	0.1176
GPLWLSV	1	3.9132	3.1026	0.1561
GPLWLSV	2	3.2506	2.8334	0.1258
GPLWLSV	3	2.7307	2.4334	0.1075
GPLWLSV	4	2.9976	2.3738	0.1216
GPLWLSV	5	1.2192	1.1111	0.053
GPLWLSV	6	0.718	0.6497	0.0313
GPLWLSV	7	2.5167	2.1156	0.1036
GPLWLSV	8	2.0709	1.9636	0.0914
GPLWLSV	9	2.1757	1.9467	0.0916
GPLWLSV	10	2.4163	2.0302	0.1007
Average		2.4009	2.056	0.0983

## Data Availability

The data used to support the findings of this study are available from the corresponding author upon request.

## References

[1] Saji N., Sato T., Sakuta K. (2014). Chronic kidney disease is an independent predictor of adverse clinical outcomes in patients with recent small subcortical infarcts. *Cerebrovascular Diseases Extra*.

[2] O’Lone E., Connors M., Masson P. (2016). Cognition in people with end-stage kidney disease treated with hemodialysis: a systematic review and meta-analysis. *American Journal of Kidney Diseases*.

[3] Bugnicourt J. M., Godefroy O., Chillon J. M., Choukroun G., Massy Z. A. (2013). Cognitive disorders and dementia in CKD: the neglected kidney-brain Axis. *Journal of the American Society of Nephrology*.

[4] Zhao Y. L., Zhang Y. H., Yang Z. K. (2019). Sleep disorders and cognitive impairment in peritoneal dialysis: a multicenter prospective cohort study. *Kidney & Blood Pressure Research*.

[5] Liang X., Wen J. Q., Ni L. (2013). Altered Pattern of Spontaneous brain activity in the patients with end-stage renal disease: a resting-state functional MRI study with regional homogeneity analysis. *PLoS One*.

[6] Chou M. C., Hsieh T. J., Lin Y. L. (2013). Widespread white matter alterations in patients with end-stage renal disease: a voxelwise diffusion tensor imaging study. *American Journal of Neuroradiology*.

[7] Chai C., Zhang M. J., Long M. M. (2015). Increased brain iron deposition is a risk factor for brain atrophy in patients with haemodialysis: a combined study of quantitative susceptibility mapping and whole brain volume analysis. *Metabolic Brain Disease*.

[8] Jiang Z. J., Zhang Y. J., Cheng Z. N. (2021). Evaluation of cognitive impairment by voxel incoherent motor imaging in patients with end-stage renal disease. *Chinese Journal of Behavioral Medicine and Brain Science*.

[9] Lu Z. X., Tu L. Y., Zu C., Zhang D. Q. (2017). Prediction of clinical variable values for Alzheimer’s disease based on brain connectivity networks. *CAAI Transactions on Intelligent Systems*.

[10] Yang M. Y., Hou W., Yang P., Zou W. B., Wang T. F., Lei B. Y. (2019). Prediction of Alzheimer’s disease clinical score based on longitudinal incomplete data combined with deep integrated regression. *Chinese Journal of Biomedical Engineering*.

[11] Zhang Y. D., Wang S. H. (2015). Detection of Alzheimer’s disease by displacement field and machine learning. *PeerJ*.

[12] Wang S. H., Govindaraj V. V., Górriz J. M., Zhang X., Zhang Y. D. (2021). Covid-19 classification by FGCNet with deep feature fusion from graph convolutional network and convolutional neural network. *Information Fusion*.

[13] Wen W., Wan Y. H., Zhang X. H., Wen Z. Y. (2021). Text feature selection based on improved CHI and PCA. *Computer Engineering & Science*.

[14] Yang S. T. (2021). Research on CET 4 score prediction model based on SVR. *Computer Knowledge and Technology*.

[15] Wang S. F., Yang X. Z., Dong Z. Y., Shi C. C. (2019). Remote sensing image change detection based on relief-PCA feature selection. *Journal of Graphics*.

[16] Zheng W. D., Li Z. G., Jia H. Z., Gao C. (2019). Prediction model of steelmaking end point based on improved Whale optimization algorithm and least square support vector machine. *Acta Electronica Sinica*.

[17] Shen L., Wang Q. T., Shi J. (2020). Single-modal neuroimaging computer aided diagnosis for schizophrenia based on ensemble learning using privileged information. *Journal of Biomedical Engineering*.

[18] Zhang X. P., Zhang X. Z. (2021). Research on photovoltaic fault diagnosis based on time domain characteristics. *Renewable Energy Resources*.

[19] Wang J. G., Chen K., Zhang W. X., Qin B. (2021). Fault diagnosis of rolling bearing based on whale optimized multi⁃core support vector machine. *Electronic Design Engineering*.

[20] Liu L. M., Li P., Chu M. X., Gao C. (2021). End-point prediction of 260 tons basic oxygen furnace (BOF) steelmaking based on WNPSVR and WOA. *Journal of Intelligent and Fuzzy Systems*.

[21] Zhe W. (2021). Optimizing BP neural network prediction model based on WOA. *International Core Journal of Engineering*.

[22] Lian Z. Y., Duan L. J., Qiao Y. H., Chen J. C., Miao J., Li M. G. (2021). The improved ELM algorithms optimized by bionic WOA for EEG classification of brain computer interface. *IEEE Access*.

[23] Zhu Z. K., Sun Y. (2021). A minimum cross-entropy multi-thresholds segmentation algorithm based on improved WOA. *MATEC Web of Conferences*.

[24] Charin C., Ishak D., Mohd Zainuri M. A. A., Ismail B., Mohd Jamil M. K. (2021). A hybrid of bio-inspired algorithm based on Levy flight and particle swarm optimizations for photovoltaic system under partial shading conditions. *Solar Energy*.

[25] Zhang Y. D., Wang S. H., Sui Y. X. (2018). Multivariate approach for Alzheimer’s disease detection using stationary Wavelet entropy and predator-prey particle swarm optimization. *Journal of Alzheimer’s Disease*.

[26] Nasri D., Mokeddem D., Bourouba B., Bosche J. (2021). A novel Levy flight trajectory-based salp swarm algorithm for photovoltaic parameters estimation. *Journal of Information and Optimization Sciences*.

[27] Balakrishnan K., Dhanalakshmi R., Khaire U. M. (2021). Improved salp swarm algorithm based on the Levy flight for feature selection. *The Journal of Supercomputing*.

[28] Isazadeh A., Tarkhaneh O., Khamnei H. J. (2018). A new hybrid strategy for data clustering using cuckoo search based on Mantegna Levy distribution, PSO and k-means. *International Journal of Computer Applications in Technology*.

[29] Sulewski P. (2021). DS normal distribution: properties and applications. *Lobachevskii Journal of Mathematics*.

[30] Loechte A., Rojas Ruiz I., Gloesekoetter P. (2021). Battery state estimation with ANN and SVR evaluating electrochemical impedance spectra generalizing DC currents. *Applied Sciences*.

[31] Li C. N., Shao Y. H., Zhao D., Guo Y. R., Hua X. Y. (2020). Feature selection for high‐dimensional regression via sparse LSSVR based on Lp‐norm. *International Journal of Intelligent Systems*.

[32] Zhang Y. T., Xi Z. T., Zheng J. H., Shi H. F., Jiao Z. Q. (2022). GWLS: a novel model for predicting cognitive function scores in patients with end-stage renal disease. *Frontiers in Aging Neuroscience*.

[33] Wang S. H., Du S., Zhang Y. (2017). Alzheimer’s disease detection by Pseudo Zernike moment and linear regression classification. *CNS & Neurological Disorders - Drug Targets*.

[34] Wang J. H., Zuo X., He Y. (2010). Graph-based network analysis of resting-state functional MRI. *Frontiers in Systems Neuroscience*.

[35] Bi X. A., Xie Y., Wu H., Xu L. Y. (2021). Identification of differential brain regions in MCI progression via clustering-evolutionary weighted SVM ensemble algorithm. *Frontiers of Computer Science*.

[36] Wang S. H., Nayak D. R., Guttery D. S., Zhang X., Zhang Y. D. (2021). COVID-19 classification by CCSHNet with deep fusion using transfer learning and discriminant correlation analysis. *Information Fusion*.

[37] Janak P. H., Tye K. M. (2015). From circuits to behaviour in the amygdala. *Nature*.

[38] Farragher J. F., Polatajko H. J., Jassal S. V. (2017). The relationship between fatigue and depression in adults with end-stage renal disease on chronic in-hospital hemodialysis: a scoping review. *Journal of Pain and Symptom Management*.

[39] Qin C., Liang Y., Tan X. (2019). Altered whole-brain functional topological organization and cognitive function in type 2 diabetes mellitus patients. *Frontiers in Neurology*.

[40] Squire L. R., Wixted J. T., Clark R. E. (2007). Recognition memory and the medial temporal lobe: a new perspective. *Nature Reviews Neuroscience*.

[41] Shackman A. J., Salomons T. V., Slagter H. A., Fox A. S., Winter J. J., Davidson R. J. (2011). The integration of negative affect, pain and cognitive control in the cingulate cortex. *Nature Reviews Neuroscience*.

[42] Chou M. C., Ko C. H., Chang J. M., Hsieh T. J. (2019). Disruptions of brain structural network in end-stage renal disease patients with long-term hemodialysis and normal-appearing brain tissues. *Journal of Neuroradiology*.

[43] Luo S., Qi R. F., Wen J. Q. (2016). Abnormal intrinsic brain activity patterns in patients with end-stage renal disease undergoing peritoneal dialysis: a resting-state functional MR imaging study. *Radiology*.

[44] Li S. M., Ma X. F., Huang R. W. (2016). Abnormal degree centrality in neurologically asymptomatic patients with end-stage renal disease: a resting-state fMRI study. *Clinical Neurophysiology*.

[45] Latora V., Marchiori M. (2001). Efficient behavior of small-world networks. *Physical Review Letters*.

[46] Kunz A., Iadecola C. (2009). Cerebral vascular dysregulation in the ischemic brain. *Handbook of Clinical Neurology*.

[47] Bi X. A., Hu X., Wu H., Wang Y. (2020). Multimodal data analysis of Alzheimer’s disease based on clustering evolutionary random forest. *IEEE Journal of Biomedical and Health Informatics*.

